# Dissemination of metabolomics results: role of MetaboLights and COSMOS

**DOI:** 10.1186/2047-217X-2-8

**Published:** 2013-05-17

**Authors:** Reza M Salek, Kenneth Haug, Christoph Steinbeck

**Affiliations:** 1 European Bioinformatics Institute, Wellcome Trust Genome Campus, Hinxton, Cambridgeshire CB10 1SD, UK; 2 Department of Biochemistry and Cambridge Systems Biology Centre, University of Cambridge, Cambridge CB2 1GA, UK

**Keywords:** Metabolomics, MetaboLights, Database, Repository, Data sharing, Standard, MSI, ISA-Tab, Curation, COSMOS

## Abstract

With ever-increasing amounts of metabolomics data produced each year, there is an even greater need to disseminate data and knowledge produced in a standard and reproducible way. To assist with this a general purpose, open source metabolomics repository, MetaboLights, was launched in 2012. To promote a community standard, initially culminated as metabolomics standards initiative (MSI), COordination of Standards in MetabOlomicS (COSMOS) was introduced. COSMOS aims to link life science e-infrastructures within the worldwide metabolomics community as well as develop and maintain open source exchange formats for raw and processed data, ensuring better flow of metabolomics information.

## Background

Metabolomics is a fast growing field, which conveys a snapshot of the metabolic dynamics or metabolic phenotype of the living organism whether healthy or in response to pathophysiological stimuli, environmental factors or disease. The number of metabolomics knowledge bases and peer-reviewed publications are rising steadily every year and there is a great need to share and disseminate metabolomics data, as the support and requirement grows from journal publishers, funding bodies and research community organisations. Within other related “-Omics” communities such as proteomics, transcriptomics and genomics it is a commonly accepted practice to share data by submitting their results to a public repository during or prior to submission of their manuscript, resulting in shared high-quality, structured data [1]. There are prerequisites within each discipline, prior to any such submission, to have agreed, community driven standards for reporting experimental data, commonly known as minimum information, using controlled vocabularies, terminologies and standard file formats to make exchangeable data more robust. It is evident that in order to have comprehensive, comparable and reproducible results you need to capture sufficient, *i.e.* minimal agreed, contextual ‘metadata’ information. Metadata itself could be quite broad; from provenance of a study material, biological and experimental metadata, to technology based information settings, protocols and parameters [1,2]. One major driving force behind such initiatives is community-sourced and agreed standards that are active, broad participation and dynamic aiming, achieving a comprehensive solution. Historically this work within the metabolomics community, after several parallel attempts, eventually lead to the formation of the Metabolomics Standards Initiative (MSI, [3,4]), culminating in several publications and recommendations after constant negotiation and coordination between stakeholders involved in development of standards within the metabolomics community. However, not many practical applications for such exercises were observed, with only few exceptions [5,6]. One major reason was due to lack of an open source, cross platform and cross species repository for capturing metabolomics experimental results while adhering to the MSI reporting requirement.

### Metabolomics repositories

In 2012 EMBL-EBI announced a general-purpose open source metabolomics repository, known as MetaboLights [7,8] to satisfy this missing gap. MetaboLights was launched at the 8th International Conference of the Metabolomics Society in Washington DC, USA. One of the main challenges was to ensure accommodation of all contextual metadata within the repository that is of interest to the community and in a practical way for users to fulfil its requirement within an acceptable time frame and effort. MetaboLights draws on the ‘Investigation/Study/Assay’ (ISA) framework, taking advantage of the modular ISA Software Suite for capturing experimental metadata and to facilitate curation at source [2]. The ISAtools suite transforms all data in ISA-tab, a highly configurable format, making it easy to conform to MSI reporting requirements and to facilitate the correct use of specific metabolomics terminologies. The metabolomics study metadata, captured using ISAtools including experimental raw files, would automatically be packaged into a zip file and uploaded to MetaboLights by the user, from which a basic ISA framework validation step will be performed. Once this validation step has been successfully passed, a unique MetaboLights identifier/accession number will be assigned to the study. After this step, with the help of the submitter, the MetaboLights curation team works toward verifying whether correct information had been captured, while adhering to MSI requirements. The curation team additionally checks for correctly annotated metadata terms linked to the most appropriate ontologies, modifying these when required. This is a manual process that requires constant communication between the curator and submitter to reach a standard agreed completion stage for the metadata captured. Metabolomics is quite a diverse field and a number of ontologies describing the metadata for metabolomics are still missing.

MetaboLights ideally requires the submitter to provide the raw experimental data using an open source format, including control samples, replicates, blank samples and any additional experiments or chemical standards used for metabolite identification. To date, within the metabolomics community there seems to be a great dependence on vendor proprietary file formats for data analysis and metabolite identification. The implementation and usage of open source files, among instrument manufacturers and the metabolomics community is not widespread. This is partially a result of the lack of a metabolomics specific exchange format and active participation of the community. While, for example there is a relatively large attendance at the annual meeting for Human Proteome Organisation’s Proteomics Standards Initiative (HUPO-PSI, [9]), until recently no such meeting has taken place for MSI, but this is about to change. The requirement for a metabolomics centric open source and standard file format resulted in the formation of COordination of Standards in MetabOlomicS – COSMOS, [8]. This consortium consists primarily of 14 European partners, but is open to the entire metabolomics community, with MetaboLights playing a central role for the coordination work. The main objective of COSMOS is to develop efficient policies ensuring metabolomics based experimental data are encoded in open standards, and tagged with a community-agreed metadata. In addition, COSMOS aims to deliver the exchange formats and terminological artifacts that are missing and are required to describe, exchange and query metabolomics experiments. Finally, COSMOS aims to develop and maintain the metabolomic based exchange formats for raw data and processed information (identification, quantification), based on the previous works of the Proteomics Standards Initiative (PSI, [9]) and to fulfil missing open standards, such as NMR Markup Language (nmrML) for capturing and disseminating Nuclear Magnetic Resonance spectroscopy data in metabolomics.

### Road ahead

There has recently been various initiatives to take metabolomics to next step, for example, the National Institutes of Health (NIH) Common Funds Metabolomics Initiatives awarded funding related to metabolomics research advancement, funding three Regional Comprehensive Metabolomics Research Cores (RCMRC) and a Data Repository and Coordination Centre (DRCC) to act as a North American hub for metabolomics related research [10]. A second round of proposals is currently under evaluation. Furthermore, the new state of the art the National Institute for Health Research (NIHR) and the Medical Research Council (MRC) Phenome Centre hosted by Imperial College London, (http://www.imperial.ac.uk/phenomecentre/) aims to analyse thousands of biofluids using metabolomics based technologies in order to facilitate discovery about how our genes interact with the environment to cause and affect the course of disease. These new initiatives as well as others by the ever-growing international metabolomics community will bring new challenges for metabolomics data handling, data analysis, knowledge curation and dissemination.

Publishers have for a long time tried to encourage making publicly available high quality metabolomics data within every published manuscript. While it is a very tedious task for publishers to judge if the data is deposited and backs up the conclusions published, this requirement is growing steadily for metabolomics studies. In contrast from a submitter point of view there is typically a concern regarding privacy of data prior to publication, so data is quite often stored behind password protected ftp sites, further complicating data exchange. With MetaboLights, other such repositories and COSMOS we are trying to address some of these issues, by providing a means for metabolomics results to be publically available to the community while considering the needs for an acceptable privacy period as requested by the submitter. We hope to further metabolomics standards via the COSMOS initiative and involvement of metabolomics stakeholders and wider community to provide open source standard file formats to capture metabolomics instrumental metadata, making the task of data submission easier, and to promote adherence and implementation of MSI recommendations for reporting metabolomics results.

## Abbreviations

COSMOS: COordination of Standards in MetabOlomics; HUPO-PSI: Human Proteome Organisation’s Proteomics Standards Initiative; ISA: ‘Investigation/Study/Assay’; MSI: Metabolomics Standards Initiative

## Competing interests

The authors declare that they have no competing interests.

## Authors’ contributions

RMS wrote the first draft with input from KH and CS, based on their experience over the last decade. All authors have read and approved the final manuscript.
